# Accessory spleen masquerading as a gastric submucosal tumor: a rare case of diaphragmatic implantation post-splenectomy

**DOI:** 10.1055/a-2740-4107

**Published:** 2025-11-28

**Authors:** Ji Zuo, Haiyang Guo, Rongmei Zheng, Qiao He, Xuemei Hou, Haorui Li, Xianfei Wang

**Affiliations:** 1 Department of Gastroenterology, Affiliated Hospital of North Sichuan Medical College, Nanchong, China; 2 117913 Digestive Endoscopy Center, Affiliated Hospital of North Sichuan Medical College, Nanchong, China; 3 Department of Cardiovascular Medicine, The Third Peopleʼs Hospital of Guangyuan, Guangyuan, China; 4 Department of Gastroscopy, Nanbu County Traditional Chinese Medicine Hospital, Nanchong, China

A 42-year-old woman with a history of splenectomy for diffuse large B-cell lymphoma presented with upper abdominal discomfort. Gastroscopy revealed a submucosal nodule in the gastric fundus, measuring 10 mm × 8 mm ( Fig. 1 **a** ). Endoscopic ultrasound (EUS) suggested that the lesion originated from the gastric fundusʼ intrinsic muscular layer, raising suspicion for a gastrointestinal stromal tumor (GIST) or another submucosal tumor ( Fig. 1 **b** ). Contrast-enhanced computed tomography (CT) showed a nodule in the gastric wall, consistent with a neoplastic process, with post-splenectomy changes evident ( Fig. 1 **c, d** ).

**Fig. 1:**
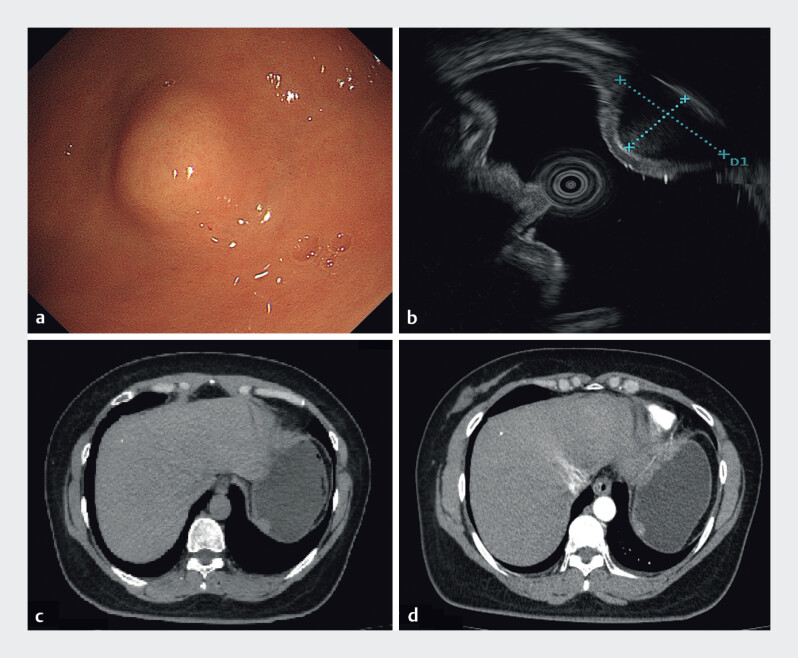
Images of the gastric fundus lesion obtained via white-light gastroscopy, endoscopic ultrasound (EUS), and abdominal pain and contrast-enhanced CT. **a** Gastroscopy revealed a submucosal nodule in the gastric fundus. **b** EUS examination showed that the lesion originated from the intrinsic muscular layer of the gastric fundus. **c** and **d** Abdominal pain and contrast-enhanced CT scans indicated a nodule in the gastric fundus wall, which was considered to represent a neoplastic lesion. Post-splenectomy changes were also observed. CT, computed tomography.

Endoscopic submucosal dissection was performed, but no lesion was identified ( Fig. 2 **a, b** ). Endoscopic full-thickness resection (EFTR) was subsequently carried out, revealing the tumor on the left diaphragm, which was completely excised ( Fig. 2 **c–f** and Video 1 ). Histopathological examination showed spindle-shaped cells, abundant sinusoids, and lymphocytic infiltration ( Fig. 3 **a** ). Immunohistochemistry was positive for CD68, Vimentin, and CD34, while other markers were negative. The Ki67 index was low ( Fig. 3 **b** ). The final diagnosis was a splenic nodule. The patient recovered without complications.

**Fig. 2:**
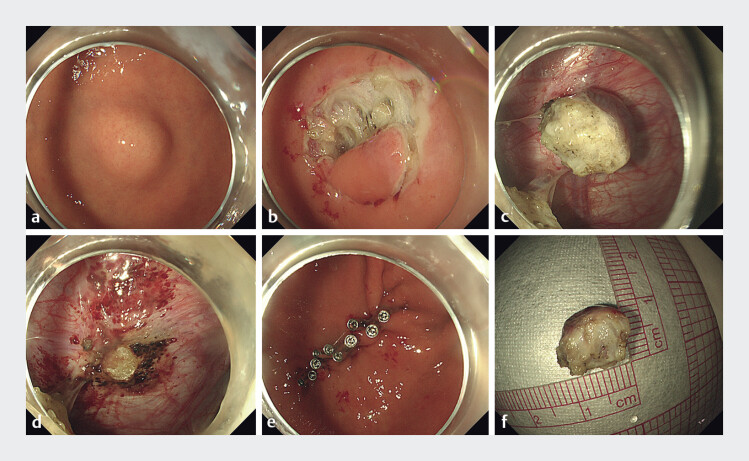
Surgical procedure of the gastric fundus lesion. **a** White-light gastroscopy revealed a submucosal nodule in the gastric fundus. **b** After endoscopic submucosal dissection (ESD), no lesion was observed. **c** Following endoscopic full-thickness resection (EFTR), a lesion was observed on the left diaphragm. **d** Postoperative changes following resection of the left diaphragm lesion. **e** The wound was sutured using an endoscopic clip. **f** The postoperative specimen.

Endoscopic full-thickness resection of a diaphragmatic accessory spleen nodule following splenectomy.Video 1

**Fig. 3:**
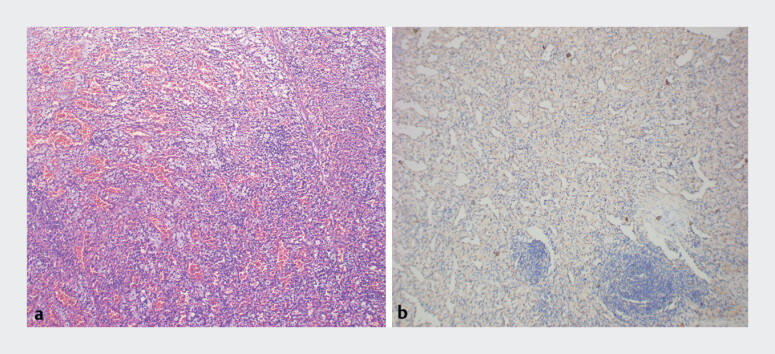
Histopathological features of the postoperative specimen. **a** H&E staining showed spindle-shaped cells, abundant sinusoids, and extensive lymphocytic infiltration. **b** Immunohistochemistry indicated a diagnosis of an accessory spleen nodule.

Accessory spleens are often found near the splenic hilum or pancreas, and rare cases involve the diaphragm 1 . Autologous splenic tissue transplantation can occur after splenectomy 2 . EUS often shows accessory spleens as hypoechoic lesions, mimicking GISTs 2 . Technetium-99 m sulfur colloid scintigraphy can aid differentiation 3 . Surgical removal is indicated when recurrence prevention or symptom relief is necessary 4 . EUS-guided FNA is valuable in preoperative diagnosis to avoid unnecessary surgery 5 .

This case highlights the need for considering ectopic accessory spleen in patients post-splenectomy with submucosal gastric lesions. A multimodal diagnostic approach is crucial for accurate diagnosis and personalized treatment.

Endoscopy_UCTN_Code_TTT_1AO_2AG_3AF

## Notes

Endoscopy E-Videos https://eref.thieme.de/e-videos
*E-Videos* is an open access online section of the journal *Endoscopy* , reporting on interesting cases and new techniques in gastroenterological endoscopy. All papers include a high-quality video and are published with a Creative Commons CC-BY license. Endoscopy E-Videos qualify for HINARI discounts and waivers and eligibility is automatically checked during the submission process. We grant 100% waivers to articles whose corresponding authors are based in Group A countries and 50% waivers to those who are based in Group B countries as classified by Research4Life (see: https://www.research4life.org/access/eligibility/ ). This section has its own submission website at https://mc.manuscriptcentral.com/e-videos .
